# Molecular characterization of *covRS* mutations in M1_UK_ *Streptococcus pyogenes*

**DOI:** 10.1002/2211-5463.70275

**Published:** 2026-06-04

**Authors:** Jarrad Pritchard, Emma‐Jayne Proctor, Jiawa Wang, Nikolas P. Johnston, Andrew Hayes, Georgia McCorkell, Haibo Yu, Jai Tree, Mark R. Davies, Mark J. Walker, Ronald Sluyter, Stephan Brouwer, Martina L. Sanderson‐Smith

**Affiliations:** ^1^ School of Science and Molecular Horizons University of Wollongong Australia; ^2^ ARC Centre of Excellence in Quantum Biotechnology University of Wollongong Australia; ^3^ The Peter Doherty Institute for Infection and Immunity University of Melbourne Australia; ^4^ School of Biotechnology and Biomolecular Sciences University of New South Wales Sydney Australia; ^5^ Centre for Superbug Solutions, Institute for Molecular Bioscience The University of Queensland QLD Australia

**Keywords:** *covRS*, M1_UK_, phosphorylation‐dependent dimerization, *Streptococcus pyogenes*, two‐component regulatory system

## Abstract

Increasingly invasive *Streptococcus pyogenes* disease has been linked to the emergence of the M1_UK_ lineage. Here, we used a murine infection model to investigate the ability of M1_UK_ to accumulate mutations in the *covRS* operon which regulates up to 15% of the genome, including multiple virulence factors. Assessing an M1_UK_ isolate carrying an Ala111Val nonsynonymous mutation showed that *in vitro,* M1_UK_ CovR^Ala111Val^ was characterized by decreased SpeB expression, increased streptolysin O and hyaluronic acid capsule expression, and resistance to neutrophil killing. While Ala111Val represents a minor biochemical change, we show that CovR^Ala111Val^ prevents phosphorylation‐dependent dimerization. Specifically, molecular dynamics simulations suggest CovR^Ala111Val^ destabilizes the interface between CovR monomers that facilitate dimerization, likely inhibiting CovR dimer‐mediated transcriptional repression and promoting altered virulence factor expression. Ultimately, this work underscores the need for ongoing epidemiological surveillance to monitor the emergence of such mutations within the already hypervirulent M1_UK_ lineage.

## Graphical abstract

Group A *Streptococcus* (GAS) acquires *covRS* mutations driving a hypervirulent bacterial state, frequently associated with invasive disease‐like necrotizing fasciitis. We demonstrate that the newly emerged M1_UK_ GAS lineage can also acquire these mutations. Notably, we demonstrate that Ala111Val destabilizes the CovR dimer interface, impairing phosphorylation‐dependent dimerization required for binding to repressor sequences, promoting derepression of key hyperinflammatory virulence factors.

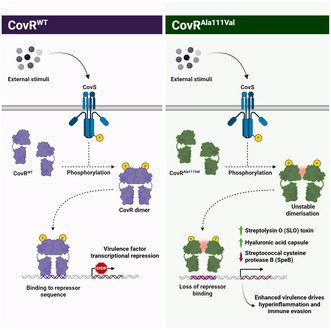

**Abbreviations**
GAS: Group A Streptococcus
iGAS: invasive Group A Streptococcus
LiKAP: lithium potassium acetyl phosphate
MD: molecular dynamics
RHD: rheumatic heart disease
RMSF: root‐mean‐square fluctuation
SLO: streptolysin O
SNPs: single nucleotide polymorphisms
SpeA: streptococcal pyrogenic exotoxin A
SpeB: streptococcal pyrogenic exotoxin B
SpeC: streptococcal pyrogenic exotoxin C
SSA: streptococcal superantigen
STSS: streptococcal toxic shock syndrome

*Streptococcus pyogenes* (Group A *Streptococcus*; GAS) is a human‐specific pathogen responsible for both superficial and invasive disease (iGAS). Superficial infections include self‐limiting conditions such as impetigo and pharyngitis. However, when GAS disseminates through the epithelial and submucosal layers, it can result in iGAS diseases including necrotizing fasciitis, bacteraemia and streptococcal toxic shock syndrome (STSS) [1]. Recurrent GAS infection can also lead to autoimmune complications such as rheumatic heart disease (RHD). The global burden of GAS disease is estimated to exceed 500 000 deaths annually, of which approximately 160 000 are attributed to iGAS [2].

Over the past decade, an increase in the incidence of iGAS has been documented globally, at least partly attributed to the emergence of the toxigenic M1_UK_ lineage [3, 4]. To date, cases of M1_UK_ have been reported in Australia, Europe, Asia, as well as North and South America [3, 4, 5, 6, 7, 8, 9]. Since its first detection in 2008 in the United Kingdom (UK) [4], M1_UK_ has become the dominant *emm*1 genotype isolated from iGAS infections in Australia and the UK [4, 6]. M1_global_ and M1_UK_ differ genetically by 27 single nucleotide polymorphisms (SNPs) and 4 indels, with M1_UK_ characterized by upregulated expression of the superantigen Streptococcal pyrogenic exotoxin A (SpeA). In addition, intermediate lineages have been reported carrying 13 of the 27 SNPs found in M1_UK_ [6]. Some M1_UK_ isolates have also been reported to possess additional virulence factors such as the phage‐encoded DNase Spd1 and superantigens Streptococcal superantigen (SSA) and Streptococcal pyrogenic exotoxin C (SpeC). The latter have been linked to immune evasion and host colonization in *emm*12 GAS responsible for a surge in scarlet fever cases in Hong Kong in 2011 [6, 10, 11, 12].

The two‐component control of virulence (CovRS) operon consists of a histidine sensor kinase (CovS) and a DNA‐binding transcriptional regulator (CovR). The CovRS operon regulates the expression of up to ~15% of the GAS genome and is typically induced during infection, upon exposure to host environmental and immune stress [13, 14]. CovR belongs to the OmpR family of bacterial transcriptional response regulators that bind to promoters and other regulatory sites as homodimeric species [15, 16]. The formation of these homodimeric species is phosphorylation dependent and occurs following phosphorylation at Asp‐53 or Tyr‐65 directly by histidine kinases like CovS or through exogenous, low‐molecular weight phosphate‐donors such as acetyl phosphate [17, 18]. Interfacing of CovR homodimers occurs through the CovR receiver domain and is facilitated through the *α*4‐*β*5‐*α*5 motif. In GAS, this motif is further stabilized by a hydrophobic patch consisting of Val89 (*α*4), Leu92 (*α*4), and Ala111 (*α*5) as well as inter‐ and intramolecular salt‐bridges that further support the hydrophobic patch [19].

Genes encoding several GAS virulence factors, including the hyaluronic acid capsule synthetic operon (*hasABC*), streptodornase I (*sda1*), streptolysin S (*sls*) and O (*slo*), interleukin‐8 cleaving enzyme (*spyCEP*), and the streptococcal cysteine protease B (*speB*), are repressed by CovR [20, 21, 22, 23, 24]. Mutations in *covRS* have been widely characterized in M1_global_ iGAS and are shown to lead to a hypervirulent phenotype distinguished by ineffective bacterial adherence to epithelial cells, bacterial survival and proliferation in the presence of immune cells, and increased haemolytic activity [14, 25, 26, 27, 28]. None of the 27 SNPs or indels that distinguish M1_UK_ from M1_global_ occur in the covRS operon. Truncations in *covS* are commonly observed in GAS harbouring *covRS* mutations and prevent phosphorylation of CovR, thereby impeding the phosphorylation‐dependent dimerization required to facilitate CovR DNA binding [29]. Mutations in *covR* occur less frequently and tend to be found at nonphosphorylation sites in the receiver domain [30]. This suggests an alternative method for loss of CovR function that does not affect the ability of CovR to be phosphorylated [30]. Furthermore, mutations in *covR* may also impact CovR dimerization and downstream repressor activity.

While the phenotypes of *covRS* mutants are well established for M1_global_ GAS, the recent identification of *covR* and *covS* mutants in M1_UK_ prompts further investigation into the potential compounding effects of *covRS* mutation in an already virulent lineage [4, 9, 31, 32, 33]. Notably, the nonsynonymous CovR^Ala111Val^ mutation was recently identified in M1_UK_, isolated from two separate Australian iGAS clinical isolates [31], with this same mutation previously associated with a fatal infection caused by the GAS *emm*81 genotype [34]. The alanine‐to‐valine substitution represents a minor biochemical change, and it is unclear how such a change affects the function and structural dynamics of CovR. In this study, we describe the prevalence of *covRS* mutation in the context of *emm*1 iGAS clinical isolates collected in Australia between 2005 and 2023, noting similar rates of mutation in M1_UK_ and M1_global_ isolates. Furthermore, we provide molecular and structural insights into how the CovR^Ala111Val^ mutation at the dimer interface disrupts dimerization, resulting in energetically unfavourable interactions that prevent repression of virulence factors, promoting phenotypes with increased invasive potential.

## Experimental procedures

### Ethics statement

The study was executed in accordance with the standards set by the Declaration of Helsinki. Experiments involving the use of human blood and neutrophils were conducted with approval from the University of Wollongong Human Research Ethics Committee (Protocol HE08/250). Informed consent was provided by healthy volunteers before donating. The animal model utilized herein was conducted with approval by the University of Queensland Anatomical Biosciences AEC (2021/AE001109).

### Bacterial strains, plasmids and culture conditions

Strains and plasmids utilized in this study are outlined in Table 1. GAS strains were routinely streak‐purified on horse‐blood Columbia agar and liquid cultures grown in Todd Hewitt broth supplemented with 1% yeast extract (THY; Edwards). *Escherichia coli* strain Bl21(DE3)pLysS was used for purification of recombinant CovR^WT^ and CovR^Ala111Val^. Plasmid selection was performed using 50 μg/mL chloramphenicol and 100 μg/mL ampicillin.

**Table 1:** Bacterial strains and isolates used in this study.

Strain or plasmid	Description	References
Strain		
*E. coli* Bl21(DE3)pLysS	F‐ *omp*T *lon hsd*SB (rB‐ mB‐) *gal dcm* (DE3) pLysS (CamR^50^, Amp^100^)	Addgene
5448	M1_global_, invasive isolate	[35]
5448AP	5448 with *covS* encoding STOP codon at amino acid (aa) 300	[35]
5448Δ*slo*	5448 Δ*slo::cat*	[36]
5448Δ*speB*	5448 with SpeB deletion	[35]
5448Δ*hasA*	5448 Δ*hasA::cat*	[25]
SP1448	M1_UK_, invasive isolate, *speA*↑	[6]
SP1567	SP1448 with *covS* encoding STOP codon at aa 35	This study
SP1584	SP1448 with *covR* encoding Asp at aa 115	This study
SP1511	M1_UK_, invasive isolate with *covR* encoding Val at aa 111	[31]
SP1512	M1_UK_, invasive isolate with covR encoding Val at aa 111	[31]
Plasmid		
pET19b+	High expression vector, T7lac promoter, adds N‐terminal 10x His‐tag, Amp^R^	Addgene
pET19b + ‐CovR^WT^	pET19b + with wild‐type *covR*	This study
pET19b + ‐CovR^Ala111Val^	pET19b + with *covR* encoding Val at aa 111	This study

### Phylogenetic screening of invasive, Australian, M1_UK_ clinical isolates

Whole genome sequence data representing 374 *emm*1 GAS isolates were collated from Queensland and Victorian public health laboratories (*n* = 370; 2005–2020 BioProject PRJNA872282), as well as New South Wales and the tropical Top End of the Northern Territory (*n* = 4 isolates; 2014–2020). This database was processed using snippy v4.6.0 (github/tseemann/snippy) with default parameters to extract SNP core alignments using the MGAS5005 reference genome (NC_007297.2). Recombinant regions within sequences were removed using Gubbins v3.4.0 [37] resulting in an alignment with a total of 792 parsimony‐informative SNPs and 1366 singleton sites. The generated alignment was used to construct a maximum‐likelihood phylogenetic tree with IQ‐TREE v2.4.0. A Hasegawa‐Kishino‐Yano substitution model with unequal nucleotide frequencies and allowing for a proportion of invariable sites (HKY + F + I) was chosen by ModelFinder according to Bayesian information criterion (BIC). The subsequent tree was visualized using iTOLv6 [38] with MGAS5005 as the outgroup. Lineage‐specific differences between the proportion of two‐component regulatory gene (*covR* and *covS*) mutations were statistically assessed using a one‐tailed proportion test (https://www.socscistatistics.com/tests/ztest/).

### *In vivo* M1_UK_ subcutaneous infection

Ethical approval: All animal procedures were conducted in accordance with the Australian Code for the Care and Use of Animals for Scientific Purposes and approved by the University of Queensland Animal Ethics Committee (approval number: 2021/AE001109).

Animals and housing: Female C57BL/6J mice (6–8 weeks old; Charles River Laboratories, Wilmington, MA, USA) were used in this study. Mice were housed in groups of five under standard conditions (12 h light/dark cycle) with *ad libitum* access to food and water.

Study design and experimental groups: Mice (*n* = 3) were assigned to a single infection group. No randomization or blinding was performed.

Experimental procedures: Mice were subcutaneously infected with approximately 1×10^8^ colony forming units (CFU) of SP1448 GAS in Dulbecco's PBS (DPBS; no calcium, no magnesium). Mice were monitored daily, and lesions harvested as described previously [20]. Isolated colonies were passaged in THY overnight with supernatants collected and SLO haemolytic and SpeB cysteine protease activity assessed to identify potential *covRS* mutants.

Animal welfare and monitoring: Animal well‐being was assessed twice daily throughout the experiment. Humane endpoints were predefined based on clinical signs of severe distress or morbidity. At 3‐days postinfection, mice were euthanized by CO_2_ inhalation using a controlled chamber.

Outcome measures, sample processing and downstream analysis: Isolated bacterial colonies were passaged overnight in Todd Hewitt yeast extract (THY) broth. Culture supernatants were collected and analysed for streptolysin O (SLO) haemolytic activity and SpeB protease activity.

### Sequencing of the *covRS* operon of animal‐passaged M1_UK_

In order to sequence the *covRS* operon, genomic DNA was extracted from overnight cultures of select GAS strains using the DNeasy Blood and Tissue Kit (Qiagen, Hilden, Germany) protocol for Gram‐positive bacteria. The *covRS* operon was amplified from gDNA using primers P1 (5′‐GCTATTCCGGTACAGGTCT‐′3) and P12 (5′‐TTGCTCTCGTGTGCCATCT‐′3) and according to the *PfuUltra* II Fusion HS DNA Polymerase protocol (Agilent) using double the recommended volume of polymerase. Amplified products were run on 1% (w/v) agarose in TAE buffer (40 mM Tris, 20 mM glacial acetic acid, 1 mM EDTA) prestained with GelRed (Biotium) for 1 h at 80 V. In parallel, PCR products were cleaned as per the QIAquick PCR Purification Kit Protocol (Qiagen, Hilden, Germany). Sanger sequencing was performed through the Australian Genome Research Facility and SNPs were detected in *covRS* via alignment against the SP1448 reference genome (GenBank: CP060267.1) in geneious prime (version 6.0, Biomatters) software. Whole genome sequencing was performed on four *covRS*‐positive mutants and two *covRS*‐negative mutants that had apparent *covRS* switching. Individual sequence reads for isolates were mapped to parent strain SP1448 (refseq version, strain ID NZ_CP060267) using breseq version 0.38.1 [39]. High confidence mutations were summarized using gdtools and individual mutations confirmed with assemblies. Assemblies were made using shovill v 1.1.0 (github/tseemann/shovill) with an underlying skesa v 2.5.1 assembler [40].

### SpeB cysteine protease activity assay

Overnight GAS cultures were centrifuged at 3220 *g* for 10 min. Culture supernatants and DPBS control were incubated alongside activation buffer [0.1 M sodium acetate (NaAc) (pH 5), 1 mM EDTA, 20 mM DTT] for 1 h at 40 °C. An equal volume of 2% (w/v) azocasein in activation buffer was added to each sample and incubated for another 1 h at 40 °C. Samples were precipitated using 2.5 volumes of 6% (w/v) TCA and immediately centrifuged at 15 000 ***g*** for 5 min. Supernatant turbidity, indicative of azocasein cleavage, was measured spectrophotometrically at 405 nm.

### SLO haemolytic assay

Overnight GAS cultures were centrifuged at 3220 *g* for 10 min. In parallel, fresh human peripheral blood was collected in lithium‐coated heparin Vacutainers (Interpath, Victoria, Australia) and centrifuged at 500 ***g*** for 10 min and subsequent plasma and buffy coats were aspirated and discarded. Remaining red blood cells (RBCs) were washed with Hanks' Balanced Salt Solution (HBSS; no calcium, no magnesium) and recentrifuged 2–3 times. RBCs were diluted to 2% (v/v) in HBSS and incubated alongside supernatants of each respective GAS strain supernatant, as well as Triton X‐100 (1% v/v) and DPBS controls. Samples were incubated for 30 min at 37 °C (5% CO_2_). Samples were centrifuged at 1000 ***g*** for 10 min to pellet RBCs, and supernatants were collected. Haemoglobin release was measured spectrophotometrically at 405 nm.

### Hyaluronic acid capsule quantification

Hyaluronic acid was purified as per [20] wherein mid‐logarithmic GAS cultures (OD_600_ 0.6) were centrifuged at 5000 ***g*** for 5 min and resuspended in 500 μL ultrapure water (UPW). An aliquot of the resuspension was serially diluted in phosphate‐buffered saline (PBS) and cultured on THY agar at 37 °C. The remaining volume (400 μL) was placed in 2 mL screw‐cap tubes alongside 1 mL chloroform and agitated for 5 min using a Mini‐BeadBeater‐8 (BioSpec Products, Bartlesville, OK, USA). Homogenates were centrifuged at 13 000 *g* for 10 min and aqueous phases containing hyaluronic acid were collected and stored at 4 °C until quantification. The quantity of hyaluronic acid was determined using a Hyaluronic Acid Test Kit (Corgenix, Broomfield, CO, USA) per manufacturer's instructions and normalized against total bacterial CFU.

### Isolation of human neutrophils

Primary human neutrophils were collected as previously described by Williams et al. [28]. Fresh human peripheral blood was collected in lithium‐coated heparin Vacutainers (Interpath) and slowly layered on an equal volume of Polymorphprep™ (ProteoGenix, Schiltigheim, France) and centrifuged at 500 ***g*** for 35 min with deceleration set to 0. Neutrophils were isolated from their respective layer and contaminating RBCs lysed hypotonically. HBSS was used to restore isotonic conditions. Neutrophils were then resuspended at the desired concentration in complete medium Roswell Park Memorial Institute (RPMI)‐1640 medium containing 2 mM L‐glutamine and 2% (v/v) heat‐inactivated autologous plasma. Neutrophils were maintained at room temperature during handling.

### Neutrophil killing assays

Following purification, neutrophil killing of GAS was performed as per Brouwer et al. [11] with minor modifications. Neutrophils were seeded into flat‐bottom 96‐well plates and infected with GAS strains at an MOI (GAS:neutrophil) of 1 : 10. Cultures were centrifuged at 500 *g* for 5 min to synchronize phagocytosis and incubated for two hours at 37 °C and 5% CO_2_. Following infection, neutrophils were lysed by the addition of 0.025% (v/v) Triton X‐100 and remaining GAS serially diluted in ultra pure water, plated onto THY agar and incubated at 37 °C. Bacterial survival was calculated as average CFU/mL in coinfected wells over CFU/mL in bacterial alone wells.

### AlphaFold CovR protein structure modelling

AlphaFold3 was used to predict the structure of CovR variants using SP1448 CovR as reference for wild‐type protein [41]. CovR^Ala111Val^ was generated by substituting valine for alanine‐111. CovR variants were modelled as homodimeric species bound to a 23‐bp *pho* Box used in the PhoB‐DNA crystal structure (1GXP). AlphaFold3 generated CovR variant PDB files were visualized using UCSF ChimeraX 1.8 [42].

### Recombinant CovR expression and purification

Cloning pET19b + ‐CovR^WT^ and pET19b + ‐CovR^Ala111Val^ was performed through Gene Universal. In brief, sequences for *covR* were sourced from the SP1448 reference genome with CovR^Ala111Val^ generated by changing GCC to GTC in the wild‐type sequence to convert Ala to Val at amino acid (aa) 111. Both wild‐type and mutant sequences were cloned between the *Nde*I and *Blp*I restriction sites of the pET19b plasmid thereby introducing an enterokinase‐cleavable 10x histidine tag on the N‐terminal end of each protein. Plasmids were transformed into *Escherichia coli* Bl21(DE3)pLysS and cultured in TYPG medium [1.6% (w/v) tryptone, 1.6% (w/v) yeast extract, 0.5% (w/v) NaCl, 0.25% (w/v) K_2_HPO_4_, and 0.5% (w/v) glucose] supplemented with ampicillin (100 μg/mL) and chloramphenicol (50 μg/mL) [43]. Bacteria were grown to OD_600_ of 0.8 at 37 °C with agitation wherein cultures were induced with 1 mM isopropyl *β*‐D‐1‐thiogalactopyranoside (IPTG) and incubated for a further four hours at 30 °C. Following incubation, bacteria were harvested via centrifugation at 5000 ***g*** and pellets stored at −20 °C. Cells were lysed per [44] using lysis buffer (50 mM NaH_2_PO_4_, 0.5 M NaCl, 0.5 mM phenylmethylsulfonyl flouride, 0.5% Tween 20; 4 mL/g pellet) supplemented with lysozyme (1 mg/mL) and incubated for 30 min on ice. Cells were further lysed through five rounds of freeze–thaw and centrifuged at 20 000 *g* for 10 min at 4 °C. Inclusion bodies were collected and solubilized using binding buffer (500 mM NaCl, 20 mM Tris‐HCl, 5 mM imidazole, 6 M urea, 5 mL/100 mL original culture volume) and incubated on ice for 1 h and subsequently centrifuged at 16 000 ***g*** (4 °C) for 30 min. Supernatant containing resolubilized protein was filtered through 0.45‐μm membranes and incubated with Ni‐NTA agarose (1 mL bed volume). Columns were washed with wash buffer (500 mM NaCl, 20 mM Tris‐HCl, 60 mM Imidazole, 6 M urea) at 4 °C and proteins eluted with binding buffer containing 500 mM imidazole. Purified protein was refolded and dialysed using storage buffer [50 mM Tris, pH 8.0, 100 mM NaCl, 1 mM EDTA, 20% (v/v) glycerol]. Protein samples were centrifuged again at 20 000 ***g*** (4 °C) for 2 min to remove any aggregates. Throughout lysis and purification, samples were routinely collected and assessed via SDS‐PAGE using Tris Glycine eXtended (TGX) stain‐free gels (Bio‐Rad, Hercules, CA, USA) and visualized using Coomassie blue R250 and Precision Plus™ Protein Dual Colour Standards molecular weight markers (Bio‐Rad).

### Circular dichroism spectroscopy

CovR secondary structures were assessed following purification and refolding using the Jasco J‐810 Spectropolarimeter (Jasco, Tokyo, Japan). CovR variants (200 μL) in 10 mM sodium phosphate buffer (pH 7.4) were loaded into a 0.1 cm pathlength cell and far‐UV circular dichroism (CD) spectral data was collected at 37 °C between 190 and 250 nm. A continuous scanning mode was set, with a bandwidth of 1 nm, response time of 2 s and data pitch of 1. Data were collected as an average of six scans, baseline corrected using buffer‐only controls and converted to molar residue ellipticity ([θ]) [45]. Determination of relative protein secondary structure abundance was performed by far‐UV CD spectra deconvolution using the bestsel software [46].

### *In vitro* CovR phosphorylation

Prior to phosphorylation, CovR samples were dialysed into phosphorylation buffer PB20 (50 mM Tris, pH 7.4, 50 mM KCl, 20 mM MgCl_2_). Phosphorylation was performed as previoulsy described [47] and initiated by the addition of phosphomimic lithium potassium acetyl phosphate (LiKAP) (MedChemExpress, Monmouth Junction, NJ, USA) to a final concentration of 50 mM, and CovR (2.85 μM) samples were left to autophosphorylate for 60 min at 37 °C.

### Mass photometry

Following phosphorylation, CovR stoichiometry was assessed using mass photometry. Samples were analysed over 10 min at a rate of 600 frames/min with an ONEMP mass photometer (Refeyn LTD, Oxford, UK). Mass photometry experiments were performed in triplicate. Data were obtained and analysed using AquireMP and DiscoverMP, version 1.2.3 (Refeyn LTD) as previously described [48]. Masses were calculated using a standard curve of the ratiometric contrast of proteins of known mass to calculate CovR masses with and without lithium potassium acetyl phosphate (Fig. S2B).

### Molecular dynamics simulations

Molecular dynamics (MD) simulations were carried out using Amber22 [49] on NVIDIA Volta or AMD Instinct MI250X GPUs. In total, four systems have been simulated, including CovR^WT^ Rec monomer, CovR^WT^ Rec dimer, CovR^Ala111Val^ Rec monomer and CovR^Ala111Val^ Rec dimer. Initial preparation of systems was done with CHARMM‐GUI [50]. The AMBER protein ff14sb force field [51], the TIP3P water model [52] and the Li and Merz 12–6 Ions models [53, 54] were applied for protein, water and ions, respectively. Molecular dynamics simulations were performed after solvating the system in a cubic box that extended at least 10 Å from the solute surface. Na^+^ and Cl^−^ counter ions were added to neutralize the system and achieve a salt concentration of 0.15 M. pKa calculations were performed using PROPKA [55, 56] to assign protonation states of ionisable residues. Simulations were performed using periodic boundary conditions (PBC) at constant temperature (303.15 K) with the Langevin algorithm (a damping coefficient of 1/ps) and at a pressure of 1.0 bar using the Monte Carlo barostat [57]. The time step was set to 2.0 fs with all covalent bonds involving hydrogens kept rigid with the SHAKE algorithm [58]. Short‐range electrostatics were calculated together with long‐range electrostatics particle mesh Ewald (PME) with a cut‐off of 9.0 Å and a PME grid size of 1.0 Å. For all systems, energy minimization (5000 steps) and 125 ps equilibration were performed first with positional restraints placed on all the protein and DNA heavy atoms (with a force constant of 1.0 kcal/ mol/Å^2^ on the protein atoms). This was followed by 2 or 4 μs production runs. Snapshots were saved every 100 ps. VMD (Visual Molecular Dynamics) [59], LOOS (Lightweight Object‐Orientated Structure Analysis) [60, 61] and MDAnalysis [62, 63] were used to analyse the trajectories.

### Free energy calculations

Free energy differences upon Ala111Val mutation on the monomer stability and the dimerization affinity were evaluated with free energy calculations. They were calculated with a double‐topology thermodynamic integration. Simulations were set up similar to the MD simulations described above. The time step was set to 1.0 fs with all covalent bonds involving hydrogens kept rigid with the SHAKE algorithm [58] except those involved in the alchemical transformation. The number of λ widows is set to 21, evenly spaced between 0 and 1. The soft‐core potential was used to avoid the endpoint catastrophe with scalpha = 0.5, scbeta = 12.0 and gti_add_sc = 5 [64]. The energy terms were saved every 5000 steps for further analysis. Additionally, the relative stabilities were predicted with FoldX (version 5.1) [65] for comparison. In the FoldX calculations, the AlphaFold predicted structures were first repaired and then free energy differences were predicted based on 10 structures of the corresponding wild‐type and mutant.

### Statistical analyses

All statistical analyses were conducted using prism software (graphpad; version 10.6.0). Statistical significance was calculated using ordinary two‐way ANOVA and Šídák's *post hoc* analysis or one‐way ANOVA using a Holm–Šídák approach for *post hoc*, where defined. *P < 0.05, ****P < 0.0001.

## Results

### M1_UK_ isolates acquire *covRS* mutations both clinically and during *in vivo* GAS infection

**Fig. 1:**
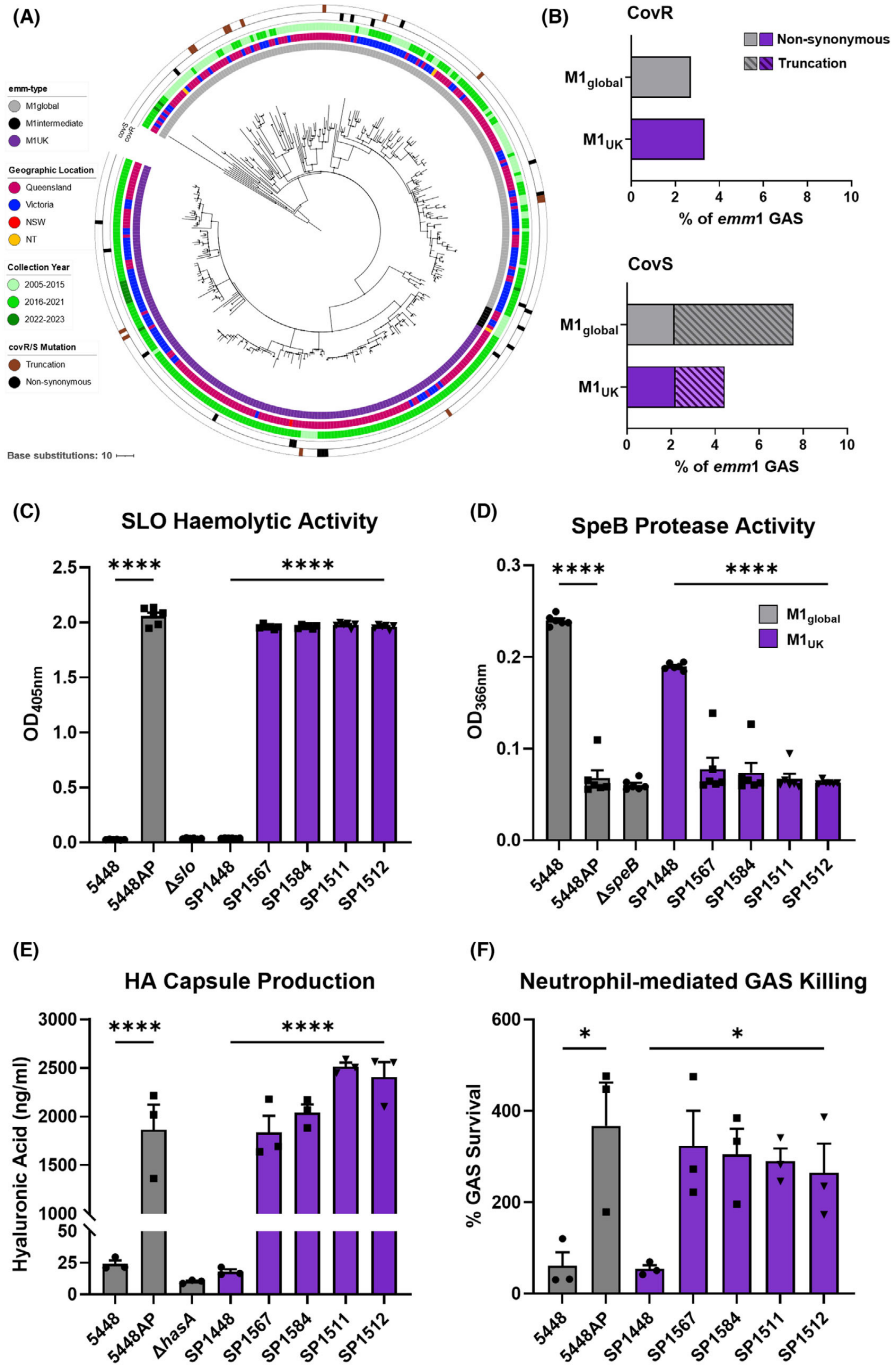
M1_UK_ acquires *covRS* mutations in clinical and *in vivo* contexts and exhibits *in vitro* phenotypes characteristic of hypervirulent M1_global_ GAS. (A) Maximum‐likelihood phylogenetic analysis built on 792 core SNPs extracted from 374 invasive *emm*1 isolates mapped to MGAS5005 (NC_007297.2). Isolates were collected in Australia (Victorian, Queensland, New South Wales and Northern Territory) between 2005 and 2023. Black circles represent branches with > 90% bootstrap support and external rings indicate isolate *emm*‐type, location, year and presence of *covR/S* mutations. (B) Frequency of *covR* and *covS* mutations in M1_global_ (grey; *covR* 5/185, *covS* 14/185) and M1_UK_ (purple; *covR* 6/180, *covS* 8/180) clinical isolates (one‐tailed proportion test: *covR P‐*value > 0.05; *covS P*‐value > 0.05). SNP type is denoted as solid bars (nonsynonymous) or diagonal bars (truncated). (C,D) SLO haemolytic and SpeB protease activity measured spectrophotometrically via haemoglobin release (405 nm) and azocasein cleavage (366 nm). (E) Hyaluronic acid capsule production in mid‐logarithmic GAS. (F) GAS survival after exposure to human neutrophils (*n* = 3 donors) assessed as percent bacterial survival in comparison to inoculum. Panels A,B show mean ± SEM (*n* = 6), representing three independent experiments. Panels C,D show pooled means ± SEM (*n* = 3 independent experiments). Animal‐passaged (AP) GAS (■), clinical GAS isolates (●), clinically derived M1_UK_ containing CovR^Ala111Val^ (▼). One‐way ANOVA was performed for all panels using the Holm‐Šídák approach for *post hoc* analyses against each lineage's wild‐type (WT) counterpart. *P < 0.05 and ****P < 0.0001. HA, hyaluronic acid, TX‐100, Triton X‐100, SLO, Streptolysin O, AP, animal passaged, SpeB, Streptococcal pyrogenic exotoxin B.

Given *covRS* mutations are commonly identified in iGAS M1_global_ isolates, we wanted to assess the *covRS* mutation frequency in iGAS M1_UK_ isolates in Australia. We performed analysis of 374 Australian GAS *emm*1 isolates using previous genomic data from Queensland and Victorian public health laboratories (*n* = 370 isolates; 2005–2020), as well as New South Wales and the Northern Territory (*n* = 4 isolates; 2014–2020) [6, 66]. Phylogenetic analysis demonstrated clear separation between M1_global_, M1_intermediate_ and M1_UK_ isolates previously defined in [6]. Mutations in *covRS* did not appear to cluster with any specific clade and were dispersed across both M1_global_ and M1_UK_ lineages (Fig. 1A). Further analysis of the *covRS* operon demonstrated that frequencies of SNPs in regulatory genes *covR* and *covS* were not significantly different between M1_global_ (2.7% and 7.6%, respectively) and M1_UK_ (3.3% and 4.4%, respectively) (one‐tailed proportion test: *covR P‐*value > 0.05; *covS P*‐value > 0.05) (Fig. 1B). Notably, mutations in *covR* were exclusively nonsynonymous, while *covS* mutations were predominantly truncations (Fig. 1B).

Next, we assessed whether *covRS* mutation could be replicated through *in vivo* passage of the invasive M1_UK_ isolate SP1448 using a subcutaneous murine infection model (Fig. S1A) [20]. Animal‐passaged (AP) isolates were screened for characteristic *covRS* mutant phenotypes wherein 35.5% exhibited increased SLO haemolytic activity, while 43.3% showed a reduction in SpeB protease activity (Fig. S1B,C). Fifteen isolates with the highest SLO activity were selected for sequencing of the *covRS* operon. Mutations in both *covR* and *covS* were identified in 40% (6/15) of selected isolates. Two representative AP M1_UK_ strains containing *covR* and *covS* mutations were selected for further characterization. These included SP1584 containing a CovR^Ala115Asp^ substitution located proximally to the previously reported Ala111Val substitution in M1_UK_ isolates SP1511/SP1512 [31] and SP1567 containing a CovS truncation at amino acid residue 35 identical to previously screened M1_UK_ clinical isolates [6]. Whole genome sequence analysis of these isolates confirmed the absence of additional mutations (Table S1). Combined, these data confirm the ability of M1_UK_ GAS to acquire mutations in *covRS* using a murine model of GAS infection and in similar locations observed in M1_global_.

To confirm the functional impact of *covRS* mutations in M1_UK_ GAS, we assessed common virulence phenotypes in clinical and AP *covRS* mutant isolates. Compared to wild‐type M1_UK_, both AP (SP1567 and SP1584) and clinically derived CovR^Ala111Val^‐mutant (SP1511 and SP1512) isolates exhibited significantly elevated SLO activity and decreased SpeB protease activity (*P* < 0.0001) (Fig. 1C,D), consistent with M1_global_ *covRS* mutants (5448AP). Additionally, significantly increased production of hyaluronic acid capsules was exhibited for both AP and CovR^Ala111Val^‐M1_UK_ isolates when compared to wild‐type M1_UK_ (*P* < 0.0001) (Fig. 1E). We next assessed GAS survival in the presence of human neutrophils. CovR^Ala111Val^ and AP M1_UK_ isolates showed resistance to neutrophil mediated killing compared to wild‐type M1_UK_ (*P* < 0.05) (Fig. 1F), as has been shown for M1_global_ *covRS* mutants.

### Ala111Val affects formation of CovR secondary structures, destabilizes CovR dimers and impacts phosphorylation‐dependent dimerization

**Fig. 2:**
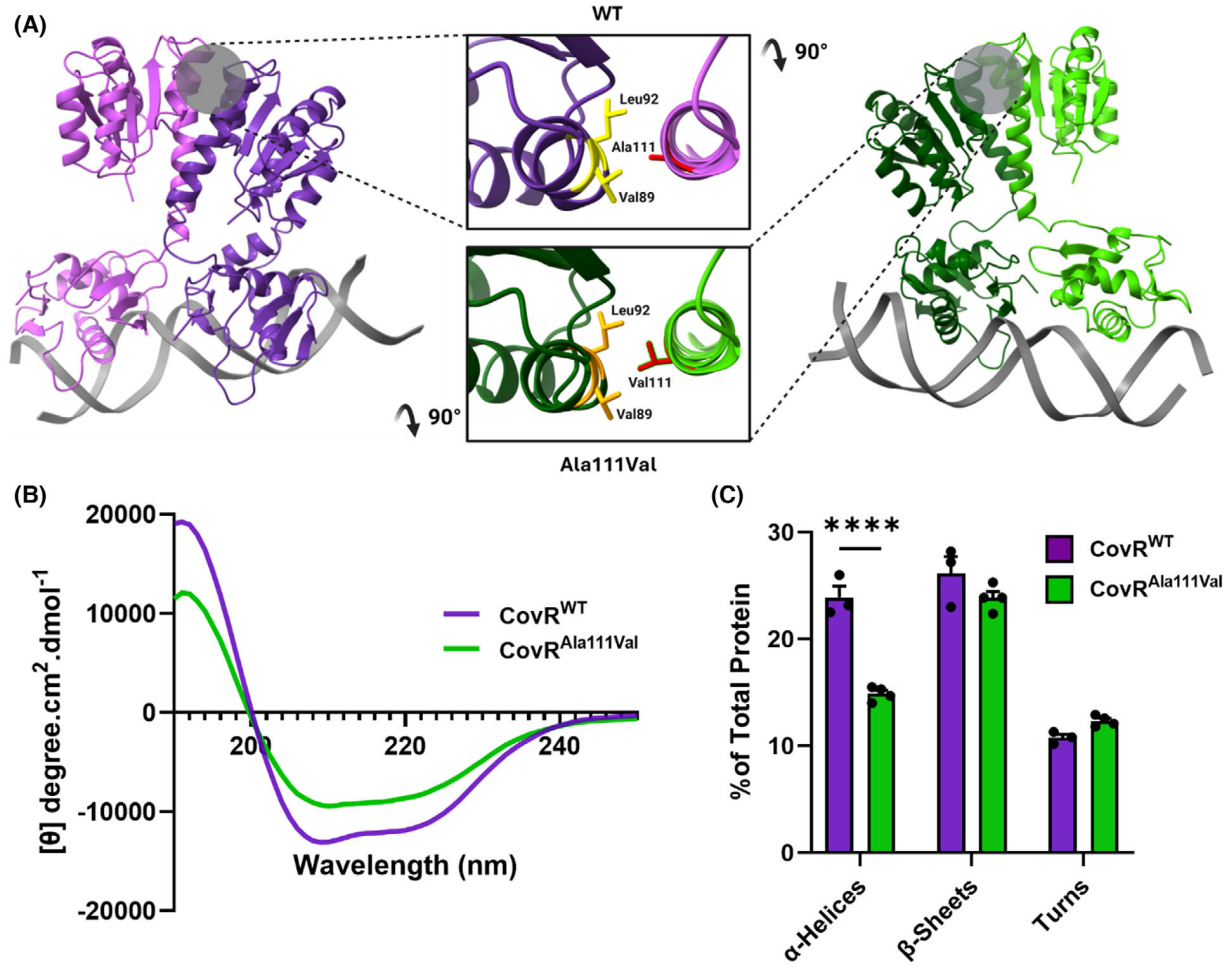
Ala111Val destabilizes CovR secondary structures. (A) AlphaFold3 modelling of CovR^WT^ (purple) and CovR^Ala111Val^ (green) as homodimeric units bound to 23‐bp *pho* Box (grey) used in PhoB‐DNA crystal structure (PDB id: 1GXP). (B) Circular dichroism (CD) spectra of recombinant CovR analysed under continuous scanning mode at 37 °C. (C) Comparative secondary structure predictions shown by deconvolution of far‐UV CD spectra in (B). Results represent the mean ± SEM of four individual scans each recorded in triplicate corrected against a buffer‐only control (B). Results represent the mean ± SEM of at least 3 deconvoluted CD spectra using bestsel software (C). Significance was determined using ordinary two‐way ANOVA and Šídák's approach *post hoc* analysis. ****P < 0.0001. Figure 2A created with BioRender.com.

Recently, an Ala111Val substitution in CovR was reported in invasive clinical M1_UK_ isolates SP1511 and SP1512, and in a previously fatal strain of *emm*81 GAS [31, 34]. Upon dimerization, the CovR homodimer is stabilized through interfacing residues and hydrophobic patches in the receiver (REC) domain, where the Ala111Val mutation is situated. An alanine‐to‐valine substitution represents a minor biochemical change, and as such, we sought to characterize how this change affects CovR structural dynamics. We first modelled the complex formed between dimeric SP1448 CovR^WT^ and CovR^Ala111Val^ subunits bound to 23‐bp *pho* box DNA using AlphaFold3. This revealed that the Ala111Val substitution occurs in the *α*4‐*β*5‐*α*5 motif, a critical region of the CovR dimer interface. Specifically, Ala111Val is positioned within the *α*4 helix and contributes to a hydrophobic patch formed alongside Val89 and Leu92 (Fig. 2A). The substitution of Ala with a slightly bulkier Val residue was predicted to destabilize CovR secondary and quaternary structures.

Firstly, we assessed secondary structures using CD spectroscopy of purified recombinant CovR^WT^ and CovR^Ala111Val^ (Fig. S2A). For both CovR variants, far‐UV CD spectra demonstrated distinct minima at 208 nm and 222 nm and maxima at 190 nm. However, CovR^Ala111Val^ showed less pronounced minima at 208 and 222 nm compared to CovR^WT^ (Fig. 2B). Using bestsel software [46], far‐UV CD spectra were deconvoluted to assess the proportion of secondary structures including *α*‐helices, *β*‐sheets and turns. There were no significant differences in the proportion of *β*‐sheets or turns between the CovR variants; however, CovR^Ala111Val^ had a significantly lower proportion of *α*‐helical structures (14.9% ± 0.29) compared to CovR^WT^ (23.9% ± 0.87) (mean ± SEM) (*P* < 0.0001) (Fig. 2C).

Next, we characterized how the Ala111Val substitution impacts CovR dimerization using *in silico*, thermodynamic integration simulation of Ala to Val in the REC domain of monomeric and dimeric forms of CovR^WT^ and CovR^Ala111Val^. The Ala111Val mutation notably destabilized dimerization of CovR by 3.8 kcal/mol (Table S2). MD simulations revealed increased root mean square fluctuation (RMSF) values, indicative of enhanced C*α* atom flexibility at interfacing residues 80–90 in one subunit of CovR^Ala111Val^ compared to CovR^WT^ (Figure S3F). Across all simulations, both CovR variants remained stable with consistent RMSD values (Tables S3 and S4, Fig. S3A–D). Combined with CD, these data suggest that the Ala111Val mutation results in energetic destabilization of CovR monomers, the CovR dimer interface region and a reduction in overall helical content, potentially affecting the ability of CovR to undergo dimerization.

**Fig. 3:**
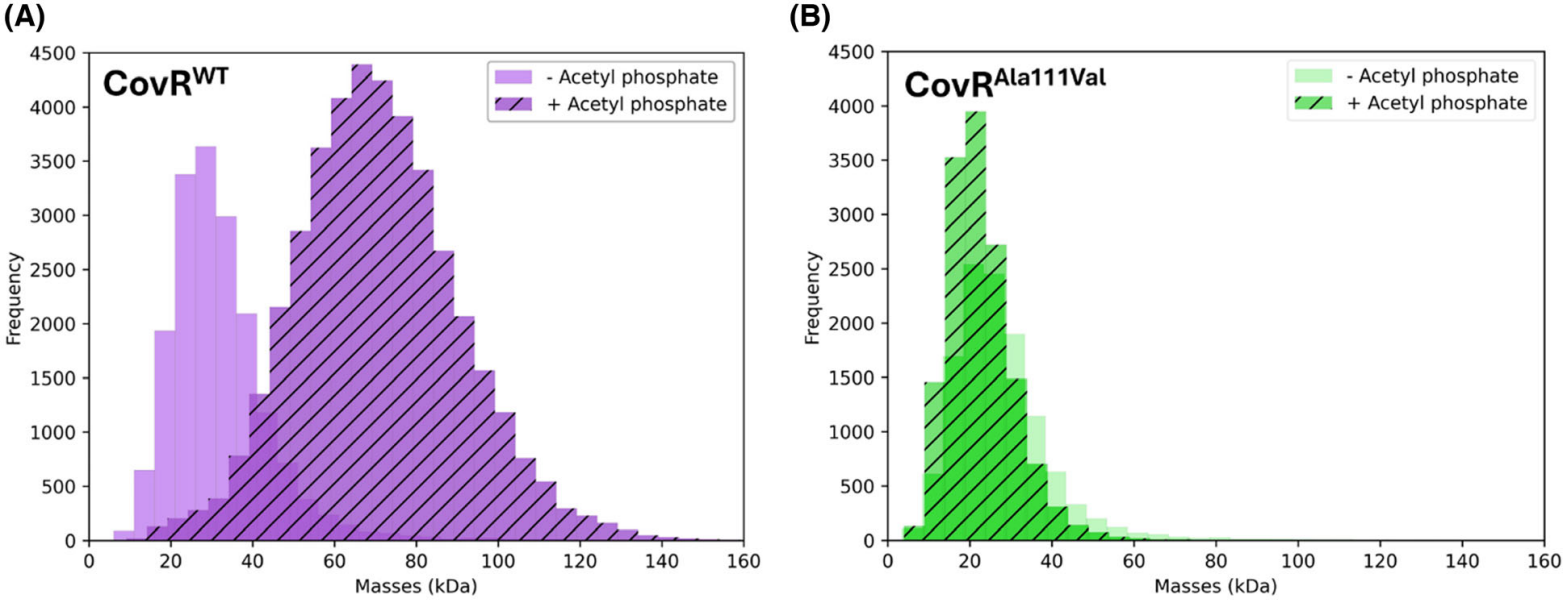
Ala111Val alters CovR stoichiometry following treatment with lithium potassium acetyl phosphate (LiKAP). CovR^WT^ (A) and CovR^Ala111Val^ (B) at 2.85 μM were artificially phosphorylated using a high‐energy phosphomimic LiKAP (28 mM) in PB20 buffer for 60 min at 37 °C and directly analysed via mass photometry. Analysis of (A) recombinant CovR^WT^ treated with LiKAP (purple, diagonal lines) demonstrates a mass distribution shift that is absent in (B) CovR^Ala111Val^ also treated with LiKAP (green, diagonal lines). Histograms represent pooled data from three independent experiments.

We next validated how Ala111Val affected the phosphorylation‐dependent dimerization of recombinant CovR using mass photometry. This was performed following treatment of CovR variants with LiKAP, a high‐energy phosphate donor that mimics endogenous bacterial acetyl phosphate that, alongside CovS, can also promote phosphorylation‐dependent dimerization [47]. Prior to phosphorylation, CovR^WT^ was shown to exist as a single monomeric species (~30 kDa), in line with its predicted mass (Figs 3A and S2A). Following incubation with LiKAP, the mass of CovR^WT^ shifted to ~65 kDa suggesting the formation of a dimeric species (Fig. 3B). However, CovR^Ala111Val^ remained monomeric before and after LiKAP treatment (Fig. 3B), confirming that the Ala111Val mutation alters the ability of CovR to undergo dimerization.

## Discussion

The resurgence of iGAS pathologies has been partly attributed to the emergence of the M1_UK_ lineage. GAS virulence is heavily regulated by several endogenous regulatory pathways, notably two‐component regulatory systems, including CovRS. Acquisition of spontaneous mutations in *covRS* during infection can generate hypervirulent strains of GAS, increasing the severity of disease [67, 68]. While mutations in *covRS* have been extensively characterized in the M1_global_ lineage, their prevalence and functional impact have been less extensively studied in M1_UK_.

In this study, we noted similar proportions of *covRS* mutation in clinical M1_global_ and M1_UK_ iGAS, and confirmed the ability of M1_UK_ GAS to acquire *covRS* mutations in a subcutaneous mouse model of infection. Mutations in *covS* were more common in both clinical and AP isolates and were composed of both nonsynonymous and frameshift mutations, as has been previously reported [29]. In the present study, we observed expected phenotypes associated with mutations in *covRS* for all M1_UK_ strains, including decreased SpeB expression, increased expression of hyaluronic acid capsule and increased SLO activity, as well as an increased ability to survive in the presence of neutrophils, as previously observed for M1_global_ GAS [20, 25, 27, 33, 69].

*covR* mutations in M1_UK_ GAS were rarer than *covS* mutations and were mainly represented by nonsynonymous mutations, consistent with previous studies [29]. Specifically, we investigated two M1_UK_ isolates from invasive infections containing a *covR* mutation encoding CovR^Ala111Val^ [31]. While the Ala111Val substitution in CovR is not novel, its impact on CovR structural dynamics remains poorly understood. Previous work demonstrated that CovR^Ala111Val^ found in a strain of *emm*81 GAS associated with rapidly progressive lethal necrotizing fasciitis exhibited poor phosphorylation‐dependent binding to virulence factor promoters [34]. However, given CovR binds to promoter sequences as a homodimer, we hypothesized that Ala111Val mediated disruption of dimerization may also contribute to *covRS* dysregulation. Given that Ala111Val resides in the hydrophobic patch of the CovR dimer interface, we proposed that its introduction may specifically alter dimer stability [19]. Molecular dynamics simulations, in conjunction with free energy calculation, demonstrated clear displacement of interfacing amino acid residues (80–90) in CovR^Ala111Val^ which likely resulted in the observed energetic destabilization at the dimer interface. However, the simulations revealed asymmetric fluctuations at the mutation site between the two monomers, with pronounced RMSF increases around the interfacing residues observed only in one subunit. This suggests that the system may have adapted to the perturbation in a nonsymmetric manner, potentially reflecting differential local responses to the mutation. We note that phosphorylation was not directly confirmed for either CovR^WT^ or CovR^Ala111Val^ following LiKAP treatment. Further investigation is therefore warranted to determine whether the secondary structure alterations observed herein influence the phosphorylation of CovR^Ala111Val^.

Herein, we have shown that the Ala111Val mutation reduces the proportion of *α*‐helices in CovR monomers. This is consistent with the fact that of the four aliphatic amino acids, valine is a weak *α*‐helix destabilizer [70]. Taken together, the position of the Ala111Val mutation in the *α*5 helix of the *α*4‐*β*5‐*α*5 interfacing motif may affect the hydrophobic patch, allowing valine‐89 and leucine‐92 greater mobility, likely contributing to the higher RMSF values observed in MD simulations. Finally, we have shown through mass photometry that phosphorylation‐dependent dimerization was significantly diminished for CovR^Ala111Val^, likely due to the aforementioned conformation changes and energetically unfavourable interactions at the CovR dimer interface.

In this study, we confirm that the M1_UK_ lineage of GAS can acquire mutations in the *covRS* regulatory system both in clinical isolates and though passage in a murine model of infection. We further demonstrate that the Ala111Val mutation in CovR disrupts monomeric secondary structures, destabilizes the dimer interface, and impairs phosphorylation‐dependent dimerization. Ala111Val has previously been shown to prevent phosphorylation‐dependent binding of CovR to key GAS virulence factor promoters requiring homodimeric CovR. We propose that this loss of binding results from a reduced ability of CovR^Ala111Val^ to dimerize. Although *covRS* mutations are relatively rare, they can significantly alter CovR and CovS protein function, leading to enhanced bacterial virulence. The identification of such mutations within the already hypervirulent M1_UK_ lineage underscores the need for ongoing epidemiological surveillance.

## Conflict of interest

The authors declare no conflicts of interest.

## Author contributions

MD, MJW, MSS and SB conceived and designed the study. AH, EJP, JP, JW, NPJ and SB performed the experiments. AH, EJP, GM, HY, JP, JW, MD, MJW, MSS, NPJ and SB analysed and interpreted the data. MJW, MSS and SB provided materials and reagents. JP wrote the original draft of the manuscript. EJP, GM, HY, JT, MJW, MRD, MSS, NPJ, RS and SB reviewed and edited the manuscript. HY, JT, MJW, MSS and SB acquired funding for respective parts of the project. EJP, HY, MD, MSS, RS and SB supervised the study.

## Supporting information


**Fig. S1.** *In vivo* M1_UK_ subcutaneous infection.
**Fig. S2.** Recombinant CovR purification and mass photometry standard curve.
**Fig. S3.** CovR molecular dynamics simulations.
**Table S1.** Identification of point mutations in M1_UK_ strain SP1448 via whole genome sequencing following animal passage.
**Table S2.** Relative stability for Ala111Val mutants based on free energy calculations.
**Table S3.** Summary of molecular dynamics (MD) simulated systems.
**Table S4.** Summary of free‐energy perturbation (FEP) dynamics simulated systems.

## Data Availability

The sequence data that support the findings of this study are openly available at NCBI BioProject PRJNA872282. The DNA sequence found within the PhoB‐DNA crystal structure (PDB: 1GXP) used in this study was obtained from the Protein Data Bank (https://www.rcsb.org/structure/1GXP). AlphaFold3 generated CovR variant PDB files are available from the corresponding author (martina@uow.edu.au) upon reasonable request. Supporting data are also available in the supporting information of this article.
