# Supplementary material for: Molecular characterization of covRS mutations in M1UK Streptococcus pyogenes
Source: FEBS Open Bio. 2026 Jun 4:10.1002/2211-5463.70275. Online ahead of print. doi: 10.1002/2211-5463.70275 (PMC13399216; doi:10.1002/2211-5463.70275)

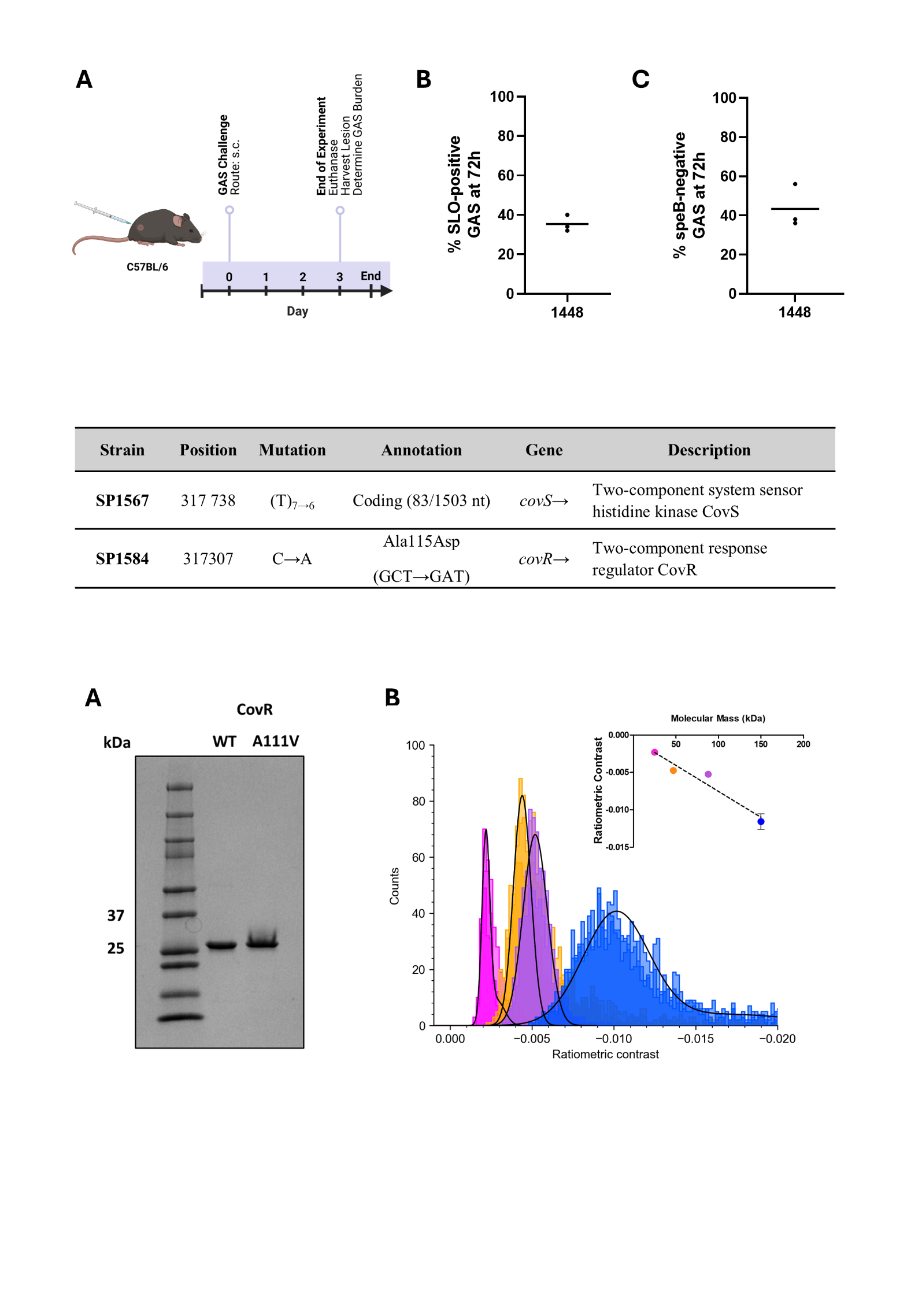


**Supplementary Figure 1: *In vivo* M1_UK_ subcutaneous infection.** (A) Representative timeline and experimental procedure for the subcutaneous, murine infection model using M1_UK_ strain 1448. (B) Percentage SLO-positive and (C) SpeB-negative GAS colonies isolated from murine skin lesions following 72-hour infection (*n* = 3 mice and 50 colonies assessed per animal). Each icon is representative of an individual mouse (B-C). Data is plotted as geometric mean and arithmetic mean for panels (B) and (C), respectively.


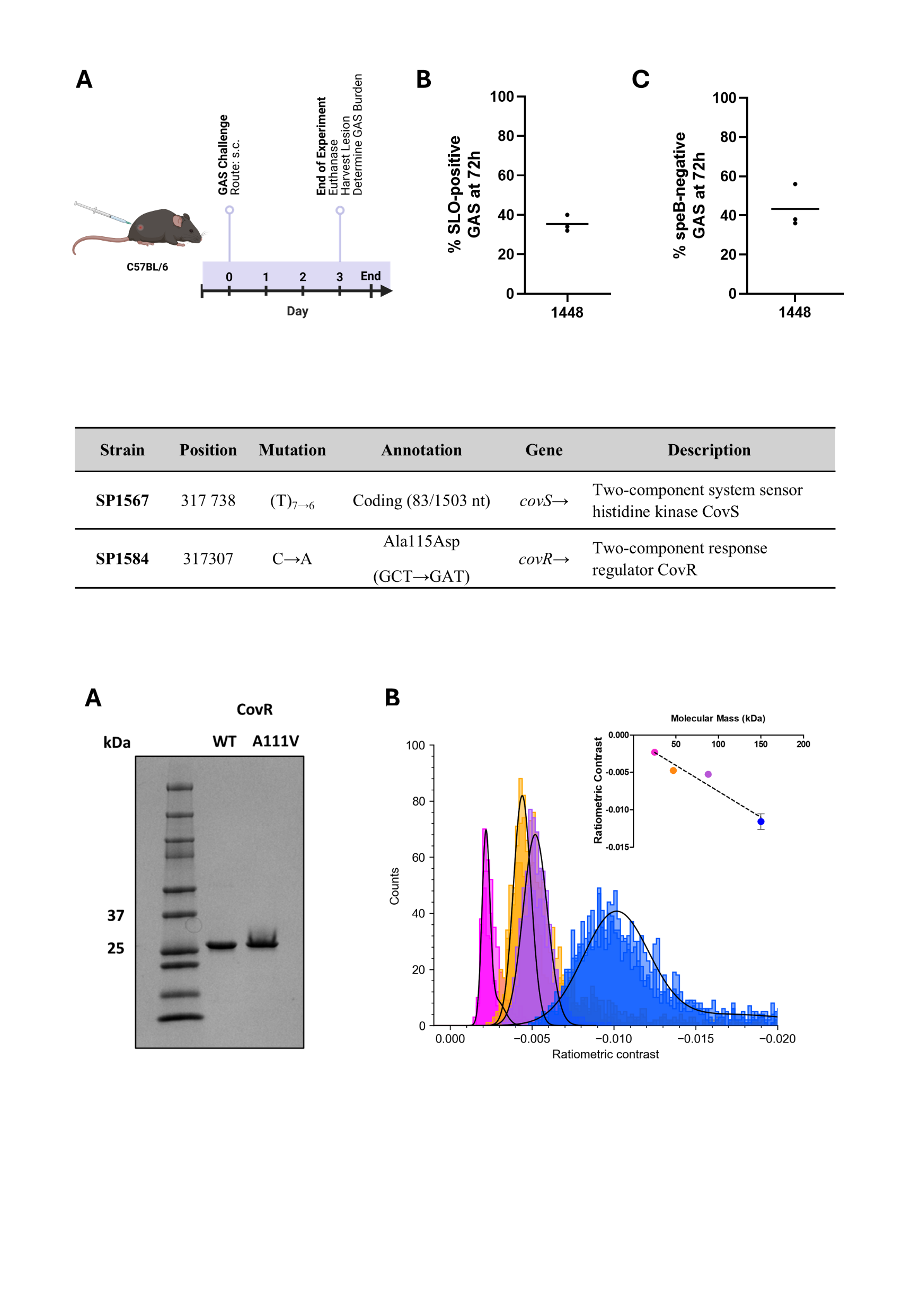


**Supplementary Figure 2: Recombinant CovR purification and mass photometry standard curve.** (A) Representative SDS-PAGE of recombinantly expressed CovR^WT^ and CovR^Ala111Val^ following refolding and centrifugation. (B) Mass photometry calibration using a standard curve of Streptococcal pyrogenic exotoxin A (29 kDa), Streptokinase (47 kDa), Plasminogen (92 kDa), and Immunoglobulin G (150 kDa) compared against measured ratiometric contrast for each protein.


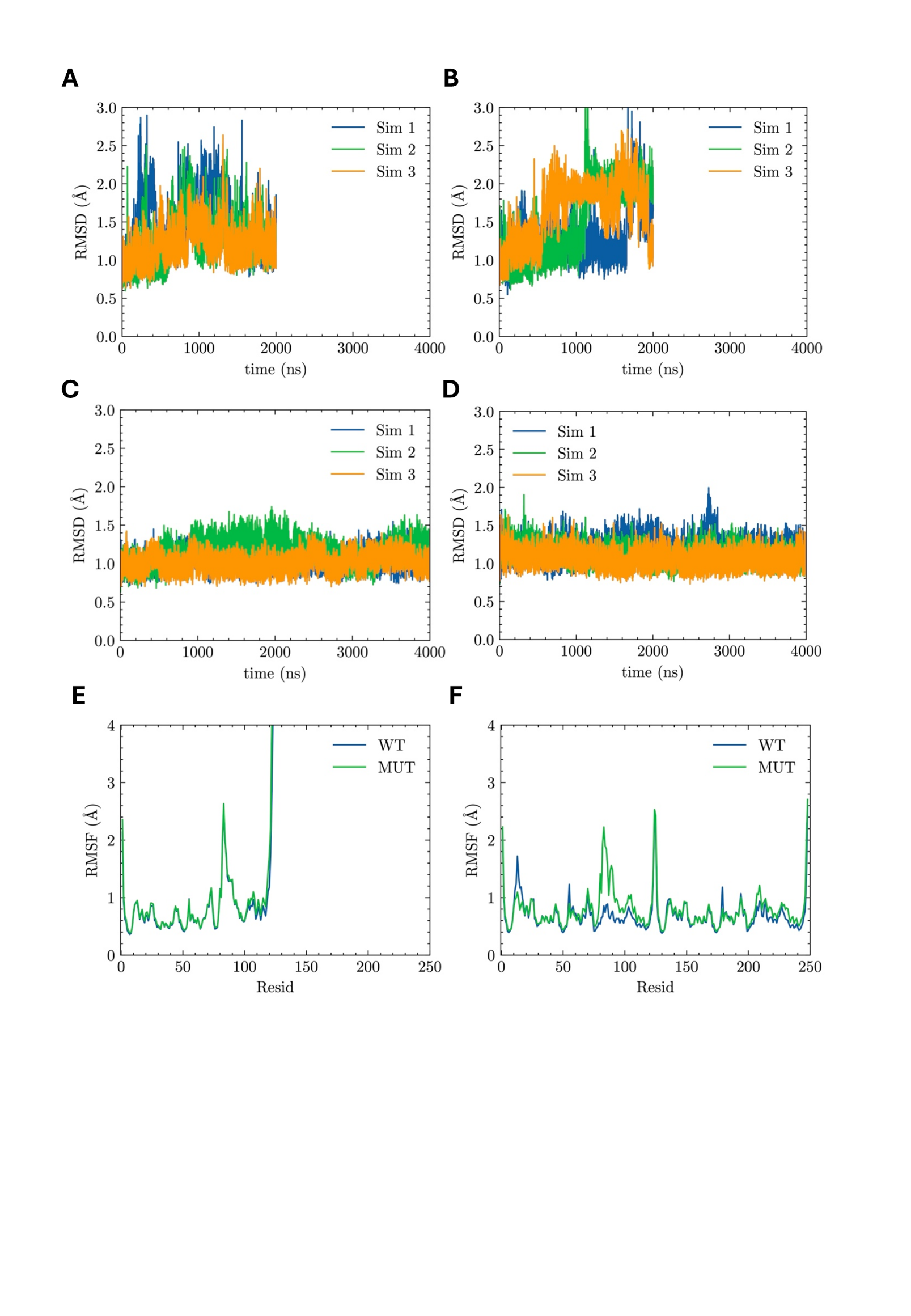


**Supplementary Figure 3: CovR molecular dynamics simulations.** The root-mean-square deviation (RMSD) of the backbone atoms with respect to the initial structure as a function of simulation time. (A): CovR Rec monomer; (B): CovR Ala111Val Rec monomer; (C): CovR Rec dimer; (D): CovR Ala111Val Rec dimer. All results are shown in triplicate. The root-mean-square fluctuations (RMSF) of the Cα atoms of monomeric (E) or dimeric (F) CovR variants in the MD simulations. (C-D) Molecular dynamics simulations of CovR receiver (REC) domains modelled as dimeric complexes demonstrate stability over the course of simulations.

**Supplementary Table 1:** Identification of point mutations in M1_UK_ strain 1448 via whole genome sequencing following animal passage.

| **Strain** | **Position** | **Mutation** | **Annotation** | **Gene** | **Description** |
| --- | --- | --- | --- | --- | --- |
| **SP1567** | 317 738 | (T)_7→6_ | Coding (83/1503 nt) | *covS*→ | Two-component system sensor histidine kinase CovS |
| **SP1584** | 317307 | C→A | Ala115Asp  (G**C**T→G**A**T) | *covR*→ | Two-component response regulator CovR |

**Supplementary Table 2:** Relative stability for Ala111Val mutants based on free energy calculations


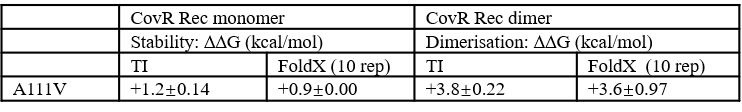


**Supplementary Table 3:** Summary of molecular dynamics (MD) simulated systems


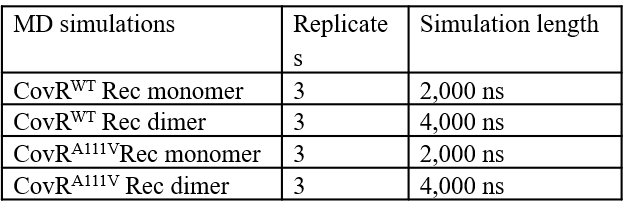


**Supplementary Table 4:** Summary of free-energy perturbation (FEP) dynamics simulated systems


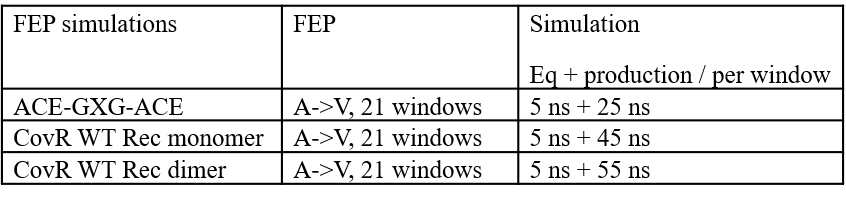

Supplement: Supplementary file 1 — Fig. S1. In vivo M1UK subcutaneous infection. Fig. S2. Recombinant CovR purification and mass photometry standard curve. Fig. S3. CovR molecular dynamics simulations. Table S1. Identification of point mutations in M1UK strain SP1448 via whole genome sequencing following animal passage. Table S2. Relative stability for Ala111Val mutants based on free energy calculations. Table S3. Summary of molecular dynamics (MD) simulated systems. Table S4. Summary of free‐energy perturbation (FEP) dynamics simulated systems. [file FEB4-9999-0-s001.docx]
